# Autonomy and focus of attention in medical motor skills learning: a randomized experiment

**DOI:** 10.1186/s12909-021-03020-z

**Published:** 2022-01-19

**Authors:** Franziska Pollok, David A. Cook, Nizamuddin Shaikh, V. Shane Pankratz, Mark E. Morrey, Torrey A. Laack

**Affiliations:** 1 Multidisciplinary Simulation Center, Mayo Clinic, Rochester, MN USA; 2 Department of Anesthesiology, University Hospital Hamburg-Eppendorf, Hamburg, Germany; 3 Division of General Internal Medicine, Department of Internal Medicine, Mayo Clinic, Rochester, MN USA; 4 Division of General Surgery, Mayo Clinic, Rochester, MN USA; 5 Internal Medicine, University of New Mexico Comprehensive Cancer Center, Albuquerque, NM USA; 6 Biostatistics Shared Resource of University of New Mexico Comprehensive Cancer Center, Albuquerque, NM USA; 7 Division of Adult Reconstruction, Department of Orthopedic Surgery, Mayo Clinic, Rochester, MN USA; 8 Department of Emergency Medicine, Mayo Clinic Multidisciplinary Simulation Center, 200 First Street SW, Rochester, MN 55905 USA

**Keywords:** Attention focus, Simulation, Autonomy, Medical motor task, OPTIMAL theory

## Abstract

**Background:**

The ‘OPTIMAL’ (Optimizing Performance Through Intrinsic Motivation and Attention for Learning) theory of motor learning suggests that autonomy, external focus of attention, and perceived competence can improve learning of simple motor tasks. The authors hypothesized that enhanced (vs. routine) autonomy and external (vs. internal) focus of attention would improve first-try performance of two medical motor tasks.

**Methods:**

The authors conducted a randomized two-by-two factorial design study with high school students as participants. Task instructions promoted either enhanced or routine autonomy, and either external or internal focus of attention. These conditions were replicated in a crossover design for two common medical tasks (chest compressions on a manikin and a Fundamentals of Laparoscopic Surgery peg transfer task). Primary outcomes were objective measures of task performance (chest compression deviation from target depth; peg transfer time with penalties for errors). Secondary outcomes included subjective perceptions of confidence, autonomy, and workload.

**Results:**

One hundred thirty-three high school students participated in this study. The primary outcomes concerning enhanced vs. routine autonomy demonstrated no statistically significant difference in either task (chest compression depth deviation: difference -0.7 mm [score range 0 to 37.5 mm]; 95% confidence interval (CI95) -3.85, 2.41; *p* = .65; peg transfer penalized time: rate ratio 1.03; CI95 0.91, 1.31; *p* = .79). The authors likewise found no statistically significant difference for external vs. internal focus of attention (depth deviation: difference 1.1 mm; CI95 -2.04, 4.17; *p* = .50; penalized time: rate ratio 0.89; CI95 0.75, 1.13; *p* = .33). The authors found no statistically significant differences for either comparison in confidence, autonomy and workload (*p* > .09; differences ranged from -0.83 to 0.79 [scale range 0 to 10]).

**Conclusions:**

First-try performance of chest compressions and peg transfer by novice learners is not significantly affected by enhanced (vs. routine) autonomy or external (vs. internal) focus of attention.

**Supplementary Information:**

The online version contains supplementary material available at 10.1186/s12909-021-03020-z.

## Background

Mastery of medical motor skills is essential for health care providers in order to deliver safe patient care. Several theories have evolved to improve our understanding of how motor skills are acquired and how learning can be optimized. One theory, the “Optimizing Performance through Intrinsic Motivation and Attention for Learning (OPTIMAL) theory”, has shown particularly promising results [1]. In this theory, two motivational factors (perceived competence and autonomy) and one attentional factor (external/internal focus of attention) play a major role in learning and retaining motor skills [1–5]. This theory has been developed and tested using a variety of simple motor tasks (e.g., balancing on a board, throwing a ball) and less often using more complex tasks (e.g., a brief gymnastics routine). In this context, ‘simple’ motor tasks describe a task with few discrete motor movements (e.g., throwing a ball at a target) while ‘complex’ tasks require a sequence of movements that must be completed in an orchestrated fashion (e.g., gymnastic routine). Instructional conditions consistent with this theory have consistently led to improved motor performance across a range of age groups and participants [2–11].

However, the OPTIMAL theory has received little attention for medical motor tasks. Since many medical procedures (e.g., laparoscopy, suturing) are more complex (involving a sequence of motor movements) than the motor tasks in which the theory has typically been tested thus far, it remains unclear whether these motivational and attentional factors will show the same beneficial learning outcomes [12]. These three factors have demonstrated improved skill developments and retention when utilized individually and also in combination [13]. The setting in which our study was conducted allowed investigation of 2 independent variables (2x2 factorial design); thus, we selected one of the two motivational factors (autonomy) and one attentional factor (focus of attention) of the OPTIMAL theory for further investigation.

### Autonomy

Autonomy describes the ability to exercise and experience control over one’s [learning] environment through self-direction [14]. A variety of studies show improved outcomes for learning motor skills when the participants are allowed greater autonomy. One explanation is that experiencing and exercising control over certain practice settings or learning environments seems to satisfy an innate psychological desire [15]. Additionally, involving the learner on how to approach the task encourages the overall motivation to learn and practice [16, 17] which could result in deeper information processing and thereby improve retention [17]. These findings hold true for both *task-related* choices [18–23] (such as the number of task repetitions) and *task-unrelated* choices [16] (such as the color of ball). Since both *task-related* and *task-unrelated* choices have been shown to promote improved learning outcomes, we chose simple, yet in the context of our study feasible, choices {chest compression side (*task-related*); wedge color (*task-unrelated*)}.

The benefits of autonomy in learning can be seen in medical education as well: A recent review on different learning techniques provides corroborating evidence that medical procedural skill acquisition is enhanced with a self-directed approach [24]. However, the direct impact of choice on medical motor skills learning remains relatively unexplored.

### External focus of attention

Another major factor described by the OPTIMAL theory is focus of attention. Directing attention in a motor task to an object or implement external to the body (e.g., a golf club or target), or the movement outcome (e.g., deviations from the midline in a balancing task), is more effective than directing attention to the internal body motion (e.g., hand position or shoulder movement). Numerous studies confirm this finding in sports [2, 25–27] and non-sports tasks [9, 28–30]. The “constraint action hypothesis” [28] offers a potential explanation for these findings: Automatic control processes that usually unconsciously regulate the fluidity of motion get constrained when learners consciously focus internally on the segmented steps of performing the movement itself. In contrast, when focusing the attention externally, interference is reduced and the motor system can operate naturally and fluidly [28]. Additionally, redirecting self-focus to the goal and/or outcome leads to more efficient goal-action coupling and facilitates improved performance [31].

Since many medical motor tasks are learned while caring for real patients in real time, educational interventions that can be implemented at the bedside, quickly and with minimal training, would be beneficial. Autonomy and attentional focus could have potentially great pedagogical impact for both learners and instructors if medical motor tasks prove to be susceptible to their influence. We hypothesized that, in the context of two medical motor tasks, novice learners would perform better under conditions that promoted enhanced (vs. routine) autonomy and external (vs. internal) focus of attention. For this study, we chose a cardiopulmonary resuscitation task (chest compressions on a manikin) and a laparoscopic box trainer basic skill (peg transfer task).

## Methods

### Overview

We conducted a randomized 2x2 factorial experiment, with crossover between two medical motor tasks (chest compressions and peg transfer) for each of the two medical tasks. The study took place in the context of a two-hour medical simulation-based activity organized to promote interest in medicine among youth at a local high school. Participants rotated among four hands-on stations, of which two were part of this study.

### Human subjects and randomization

**Fig. 1:**
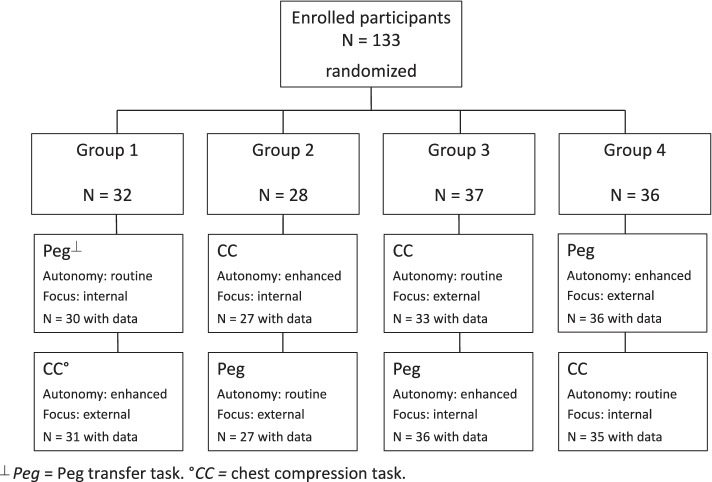
Trial flow of 2x2 factorial design. ^⏊^ *Peg* = Peg transfer task. °*CC =* chest compression task

All participants were high school students. Parents or guardians of each student gave informed consent before participation. All students participating in the activity were eligible to participate in this study. Participants were randomly assigned to one of four groups (see Fig. 1) using a list of numbers randomly generated in Excel® (Microsoft, Redmond, USA) and everyone in a given group received the same intervention conditions, such that conditions were assigned randomly. The study was classified as exempt by the Mayo Institutional Review Board.

### Tasks

#### Chest compression task

The participants watched an approximately 2-minute introduction video focusing on the importance and correct technique to administer chest compressions, consistent with current American Heart Association (AHA) guidelines [32]. Instructors then emphasized the importance of adequate depth and rate using a standard script, followed by additional brief instructions specific to the independent variables (detailed below). Each participant then performed chest compressions (without ventilation) for 45 seconds on a SimMan®3G manikin (Laerdal Medical, Stavanger, Norway) without any feedback.

#### Peg transfer task

The peg transfer task was adapted from the Fundamentals of Laparoscopic Surgery (FLS) system (SAGES Fundamentals Division, Los Angeles, USA), a widely accepted tool for training and assessment in laparoscopic surgery [33, 34]. The participants watched an approximately 2-minute instruction video on how the task was to be performed. The instructors gave a brief explanation of the instruments (graspers) for all groups, followed by brief instructions specific to the independent variables (detailed below). They were then asked to transfer, in a defined sequence, 3 wedges from numbered pegs (1, 2, 3) on the left side of a pegboard to corresponding numbered pegs on the right side.

### Independent variables

With guidance from an expert in the OPTIMAL theory and related research, we planned our study and interventions. Our study evaluated two independent variables, each with two conditions. The two motor tasks were identical for all groups in goals and techniques; the tasks varied only in differences in the verbal instructions concerning autonomy and the focus of attention (verbatim instructions are reported in the Additional file 1: Appendix). Each participant completed one task with routine autonomy and the other with enhanced autonomy, and likewise completed one task with internal focus and the other with external focus. The autonomy intervention (choice or assignment as noted below) occurred first, followed immediately by the attention intervention (instructions to focus externally or internally).

#### Autonomy

As noted above, both task-related and task-unrelated choices have been shown to enhance performance; for this study we selected one of each. For chest compressions, participants in the enhanced autonomy condition were given the choice of standing on the right or left side of the manikin (a task-related choice). For peg transfer, they could choose the wedge color (orange or green) and the sequence (1-2-3 or 3-2-1) in which to transfer the wedges (a task-unrelated choice).

Participants in the routine autonomy condition were assigned the task condition (i.e., left or right side for the chest compressions; color and sequence for peg transfer). These conditions were assigned such that the number of participants choosing and assigned to each task condition was similar (i.e., yoked assignment).

#### Focus of attention

Participants in the external focus condition were instructed to concentrate on the manikin's chest or the grasper tips. After 20 seconds of chest compressions or after the first completed wedge transfer, the instruction to focus attention on the manikin’s chest or grasper tip was repeated.

Participants in the internal focus condition were instructed to concentrate on their hands. This instruction was repeated after 20 seconds of chest compressions or after the first wedge transfer.

### Instruments and outcomes

Outcomes included task-specific objective measures of performance, and self-reported perceptions of confidence, autonomy and workload.

#### Chest compression performance measures

Depth and rate were computer recorded from the manikins’ internal sensors. The primary performance outcome was “depth deviation,” specified as the average distance of compressions outside the target range defined by the AHA [32]. Compression depth of 50 – 60 mm (2 – 2.4 inches) was scored as zero deviation, while compressions < 50 mm (< 2 inches) or > 60 mm (>2.4 inches) were scored as the absolute value of distance outside the target range (i.e., both 45 mm and 65 mm would be scored as 5). Secondary performance outcomes included “rate deviation,” scored similarly to “depth deviation” (relative to a target range of 100-120 compressions per minute), and a composite score “chest compression success” defined as the proportion of time for which target depth and target rate were achieved simultaneously.

#### Peg transfer performance measures

Live performance was assessed by one of five independent and experienced raters who are routinely involved in assessing the FLS peg transfer task in surgical residents. Performance was assessed using a score sheet modeled after the official FLS guidelines [35]. It was not possible to blind raters, who also served as station supervisors. The primary outcome was “penalized time,” a composite score defined as total task time plus a time penalty for errors. The time penalty for each error was one-sixth of the average error-free time (i.e., average time for all participants who performed the task without error). An error (and corresponding penalty) was counted if a wedge was dropped, a double penalty was assigned for wedges dropped out of the field of vision. Secondary performance outcomes included total task time (without penalty) and the number of error events (dropped wedge, instrument tip out of vision).

#### Perceived confidence, autonomy, and workload

As secondary outcomes, we assessed self-reported motivation (in the domains of confidence [2 items at different levels of task challenge] and autonomy [1 item]) and perceived workload (1 item) using a four-item questionnaire at the end of each task (see Additional file 1: Appendix for verbatim wording).

### Data analysis

All participants were analyzed according to their initial group randomization (intent-to-treat). We planned to first test for interaction between the two design factors (focus and autonomy) and then proceed with comparison of main effects within factors only if no interaction was found. We examined the residuals from each linear model to evaluate whether the data met required assumptions. All reported linear model analyses were performed using data that adequately conformed to the required assumptions on either the original or log-transformed scale.

Chest compression outcomes were analyzed using adjusted models to account for potential confounders for age, sex and manikin to evaluate the potential impact of the two experimental factors of autonomy and attentional focus. Outcomes for depth deviation and rate deviation were analyzed using linear models. Overall chest compression success was analyzed with negative binomial regression models on the count of compressions in the target depth and rate, offset by the total number of compressions.

Peg transfer outcomes were analyzed using adjusted models to account for potential confounders of age, sex, and dominant hand. Outcomes for time (both with and without error penalty) were analyzed on the logarithmic scale using linear models. The number of times a wedge was dropped and number of times it was out of the field of vision was analyzed using generalized linear models approaches with negative binomial distribution. The number of times an instrument was out of the field of vision was analyzed using generalized linear models approaches with Poisson distribution.

Perceived confidence, autonomy, and workload outcomes were analyzed using linear models adjusted to account for potential confounders of age and sex. A two-sided alpha of 0.05 was used to test for significance. We estimated power to detect differences between groups of the 2x2 factorial design, for analyses that would use outcomes data from both time points within a person. Using a simulation study that varied the within-person correlation across a plausible range, we estimated that 120 participants would provide 90% power to detect a difference of 0.5 standard deviations even allowing for higher-than-anticipated within-person correlations of 0.7. To avoid bias, the statistician was blinded to the different conditions until the general analysis was completed. Data analysis was done with SAS version 9.4 (SAS Institute, Cary, NC).

## Results

### Participants

All 133 participants completed at least one station; after attrition due to data collection difficulties, we had data for 126 participants for chest compressions and 129 participants for peg transfer (see trial flow in Fig. 1). One hundred twenty-nine participants provided demographic data (Table 1).

**Table 1:** Participant demographics

Characteristic	Response	Group 1 ***N***=31	Group 2 ***N***=27	Group 3 ***N***=36	Group 4 ***N***=35
Age, mean (SD)	Years	15 (1.0)	14.7 (1.0)	14.8 (0.9)	14.8 (1.0)
Sex, No. (%)	Male	15 (48%)	13 (48%)	17 (47%)	9 (26%)
Grade in school^a^	9	15/30 (50%)	18/27 (67%)	24/36 (67%)	18/32 (56%)
	10	11/30 (37%)	4/27 (15%)	5 (14%)	9/32 (28%)
	11	2/30 (7%)	2/27 (7%)	4 (11%)	2/32 (6%)
	12	2/30 (7%)	3/27 (11%)	3 (8%)	3/32 (9%)
Dominant hand	Right	27 (87%)	26 (96%)	35 (97%)	33 (94%)

^a^some students did not provide an answer

### Autonomy: enhanced vs. routine

We found no statistically significant differences in the primary outcome between enhanced and routine autonomy for either chest compressions or peg transfer (see Table 2). For chest compressions, the depth deviation (smaller values reflect better performance) was 15.3 mm for enhanced autonomy and 16.0 mm for routine autonomy (difference -0.7 mm [score range 0 to 37.5 mm]; 95% confidence interval [CI95] -3.85, 2.41; *p* = .65). For peg transfer, the penalized time (smaller values reflect better performance) was 1.53 minutes (i.e., 1 minute 32 seconds) for enhanced autonomy and 1.48 minutes for routine autonomy (rate ratio 1.03; CI95 0.81, 1.31; *p* = .79).

**Table 2:** Performance outcomes for autonomy

	N	Enhanced autonomy	Regular autonomy	Difference [D] or rate ratio [RR] (CI95)^c^; ***p***
Chest compressions				
**Depth deviation** in mm; mean [SE]^a^	126	15.3 [1.2]	16.0 [1.1]	D: -0.7 (-3.85, 2.41); *p*=.65
**Rate deviation** in bpm^b^; mean [SE]	126	5.4 [2.8]	7.4 [2.6]	D: -2.0 (-9.54, 5.2); *p*=.57
**Overall success;** % of time (CI95)	126	3.5 (1.5, 8.2)	3.3 (1.4, 7.9)	RR: 1.06 (0.3, 3.62) *p*=.94
Peg transfer				
**Penalized time** In minutes; mean (CI95)	129	1.53 (1.30, 1.79)	1.48 (1.24, 1.77)	RR: 1.03 (0.81, 1.31); *p*=.79
**Raw time** In minutes; mean (CI95)	129	1.38 (1.18, 1.61)	1.39 (1.17, 1.66)	RR: 0.99 (0.78, 1.25); *p*=.93
**Wedge drops;** mean (CI95)	129	0.75 (0.52, 1.08)	0.70 (0.47, 1.06)	RR: 1.07 (0.62, 1.84); *p*=.81
**Instrument out of vision**; mean (CI95)	129	0.35 (0.23, 0.53)	0.10 (0.04, 0.23)	RR: 3.5 (1.4, 9.03) *p*=0.002

Rate ratio is reported (instead of difference between means) for analyses conducted using log-transformed data

^a^ *SE* standard error

^b^ *Bpm* beats per minute

^c^ *CI95* 95% confidence interval

We found a statistically significant difference in one secondary outcome of performance: The error rate for instruments out of field in peg transfer was 0.35 errors per person for enhanced autonomy, and 0.1 errors for routine autonomy (rate ratio 3.55; CI95 1.4, 9.03; *p* = .002). We found no statistically significant difference in the remaining secondary outcomes of performance, or in any measures of confidence, autonomy or workload (see Tables 2 and 4).

### Focus of attention: external vs. internal

We found no statistically significant differences in the primary outcome between external and internal focus of attention, for either chest compressions or peg transfer (see Table 3). For chest compressions, the depth deviation was 16.2 mm for external focus, and 15.1 mm for internal focus (difference 1.1 mm; CI95 -2.04, 4.17; *p* = .50). For peg transfer, the penalized time was 1.42 min for external focus and 1.59 min for internal focus (rate ratio 0.89; CI95 0.7, 1.13; *p* = .33). We found no statistically significant differences in secondary outcomes of performance, or in any measures of confidence, autonomy or workload (see Tables 3 and 4).

**Table 3:** Performance outcomes for focus of attention

	N	External focus	Internal focus	Difference [D] or rate ratio [RR] (CI95)^c^; ***p***
Chest compressions				
**Depth deviation** in mm; mean [SE]^a^	126	16.2 [1.1]	15.1 [1.1]	D: 1.1 (-2.04, 4.17); *p*=.50
**Rate deviation** in bpm^b^; mean [SE]	126	8.5 [2.5]	4.3 [2.7]	D: 4.2 (-3.14, 11.61); *p*=.26
**Overall success;** % of time (CI95)	126	2.1 (0.9, 4.9)	5.6 (2.5, 12.6)	RR: 0.37 (0.12, 1.21) *p*=.10
Peg transfer				
**Penalized time** In minutes; mean (CI95)	129	1.42 (1.20, 1.68)	1.59 (1.34, 1.89)	RR: 0.89 (0.7, 1.13); *p*=.33
**Raw time** In minutes; mean (CI95)	129	1.30 (1.10, 1.53)	1.48 (1.25, 1.74)	RR: 0.88 (0.7, 1.1); *p*=.27
**Wedge drops;** mean (CI95)	129	0.69 (0.47, 1.03)	0.76 (0.52, 1.11)	RR: 0.91 (0.53, 1.57); *p*=.72
**Instrument out of vision**; mean (CI95)	129	0.15 (0.07, 0.33)	0.23 (0.13, 0.41)	RR: 0.66 (0.26, 1.66) *p*=.36

Rate ratio is reported (instead of differences between means) for analyses conducted using log-transformed data

^a^ *SE* standard error

^b^ *Bpm* beats per minute

^c^ *CI95* 95% confidence interval

**Table 4:** Self-reported outcomes of perceived confidence, workload and autonomy

**Autonomy**	**Enhanced**	**Routine**	**Difference (CI95)**^b^**;** ***p*-value**
Chest compressions			
Confidence, moderate goal Mean [SE]^a^	7.11 [0.27]	7.22 [0.25]	-0.11 (-0.85, 0.63); *p* = .77
Confidence, challenging goal Mean [SE]	6.17 [0.30]	6.13 [0.28]	0.04 (-0.78, 0.85); *p* = .93
Workload Mean [SE]	6.36 [0.34]	5.57 [0.31]	0.79 (-0.12, 1.71); *p* = .09
Autonomy Mean [SE]	8.25 [0.18]	8.30 [0.17]	-0.05 (-0.55, 0.44); *p* = .81
Peg			
Confidence, moderate goal Mean [SE]	5.82 [0.35]	6.65 [0.39]	-0.83 (-1.86, 0.2); *p* = .11
Confidence, challenging goal Mean [SE]	4.44 [0.33]	4.65 [0.37]	-0.21 (-1.18, 0.77); *p* = .67
Workload Mean [SE]	6.40 [0.26]	6.37 [0.29]	0.03 (-0.75, 0.82); *p* = .92
Autonomy Mean [SE]	7.75 [0.21]	7.94 [0.24]	-0.19 (-0.82, 0.45 *p* = .55
**Focus**	**External**	**Internal**	**Difference (CI95);** ***p*-value**
Chest compressions			
Confidence, moderate goal Mean [SE]	7.09 [0.26]	7.25 [0.26	-0.16 (-0.9, 0.57); *p* = .66
Confidence, challenging goal Mean [SE]	6.07 [0.29]	6.23 [0.29]	-0.16 (-0.98, 0.65); *p* = .69
Workload Mean [SE]	5.70 [0.32]	6.23 [0.33]	-0.53 (-1.45, 0.38); *p* = .24
Autonomy Mean [SE]	8.12 [0.17]	8.43 [0.17]	-0.31 (-0.79, 0.19); *p* = .22
Peg			
Confidence, moderate goal Mean [SE]	6.09 [0.37]	6.37 [0.37]	-0.28 (-1.31, 0.75); *p* = .59
Confidence, challenging goal Mean [SE]	4.57 [0.35]	4.52 [0.35]	0.05 (-0.93, 1.02); *p* = .92
Workload Mean [SE]	6.24 [0.28]	6.53 [0.28]	-0.29 (-1.07, 0.5); *p* = .46
Autonomy Mean [SE]	8.10 [0.21]	7.59 [0.23]	0.51 (-0.13, 1.14); *p* = .11

Answers given in Likert type response options ranging from 0 (not confident at all, very low, strongly disagree) to 10 (very confident, very high, strongly agree)

^a^ *SE* Standard error

^b^ *CI95* 95% Confidence interval

### Interaction between autonomy and attentional focus

In this 2x2 factorial design, we tested for the presence of interactions between the design factors and the outcomes measured from the two medical motor tasks. We found no statistically significant differences in the primary or secondary outcomes on the interaction of autonomy and attentional focus for either chest compression or peg transfer task (*p* > .10). We therefore limited our attention to the main effects of the intervention factors on the study outcomes, as above.

## Discussion

We hypothesized that novice learners would perform better under conditions that promoted enhanced autonomy and an external focus of attention during performance of two medical motor tasks. We found no significant differences for all primary and most secondary outcomes, including objective measures of task performance and subjective measurements of confidence, autonomy and workload.

### Limitations and strengths

The participants were high school students and therefore younger than most healthcare learners. However, we believe that our findings can generalize to novice medical learners and note in particular that the peg transfer task is novel to most medical students. Moreover, the chest compression task is an important skill for non-healthcare learners [36, 37]. Although all participants volunteered for this optional half-day activity, it is nonetheless possible that some did not perform to the best of their ability. We chose a *task-unrelated* choice for the peg transfer autonomy intervention, and it is possible that a *task-related* choice would have had a stronger effect. Strictly speaking, this is a study of motor skills performance rather than motor skills learning, since performance on the first and only completion was used in assessing the performance outcomes. The students in a given group were able to observe their peers’ performance, which might have influenced performance in those late in the rotation. However, everyone in a given group received the same autonomy or attention condition, which would minimize the confounding impact. Strengths include the randomized crossover design, use of two distinct medically-relevant tasks, ample sample size (and confidence intervals that exclude educationally significant effects), and objective measurement of performance using outcomes reported in previous research [34, 38, 39].

### Integration with prior work

Our findings contradict our hypotheses and previous research on OPTIMAL theory and motor task performance (see review by Wulf et al. [1]). We speculate several reasons why our hypotheses were not confirmed. First, and in our mind most likely, it is possible that first try performance of motor tasks is less influenced by these attentional and motivational factors than are repeated performance and learning. Second, it is possible that complex tasks are less impacted by these factors than are the simple tasks used in most prior research in the OPTIMAL theory. If true, this has broad implications for application of this theory to medical motor tasks (which are usually complex). Third, our interventions may have been too weak or misaligned with the underlying conceptual construct, or our assessment might have been insensitive to changes. However, our study design, interventions, and assessments were developed in close collaboration with an expert in the field of OPTIMAL theory, which makes this less likely as the sole explanation for our negative findings.

### Implications for educators and future research

We highlight the following implications for educators and future research. First it seems that OPTIMAL theory, and corresponding instructional principles, may not apply in the context of first-try (single repetition) performance in medical motor skills learning. Naturally, this merits confirmation in future research. However, if confirmed, it would underscore a novel insight with potentially broad implications for medical motor task learning – namely that the conditions of learning differ for single vs. repeated experience with a given task. Future research to elucidate this issue might include multiple practice trials and subsequent retention tests.

Our negative findings for secondary outcomes have implications that extend beyond first-try performance. Our interventions did not show any demonstrable impact on confidence (i.e., for performance in future attempts) or perceived autonomy (which should have been affected even if performance was not). This suggests that the impact of these interventions on repeated performance and learning would be limited. Again, this merits confirmation in future research.

Finally, these findings highlight several directions of promise for future research. First, future work might elucidate the issue of first-try vs. repeated performance, by comparing performance on first vs. subsequent repetitions. Including assessments of learning retention would also be ideal. Second, future investigations might try other approaches to operationalize OPTIMAL theory into stronger yet still practicable interventions relevant to medical motor task performance and learning. Third, research might explicitly contrast (i.e., using the same participants, the same interventions, and comparable outcomes) tasks of varying complexity (such as throwing a ball and performing a peg transfer task), to investigate the question of whether the OPTIMAL theory differentially applies to simple vs. complex tasks or medical vs. non-medical motor skills. Research might similarly explore how the theory applies to learners at different levels of training. These and other studies will refine our understanding of the principles that govern medical motor skills learning, and enable more effective and efficient instructional design for procedural skills education.

### Conclusion

Our study did not show significant improvement in medical motor skills performance when adopting an external focus of attention and/or enhanced autonomy. Future research implementing (for example) repeated performance assessments, stronger interventions or different task complexity will help to better understand influences in medical motor skill learning.

## Supplementary Information

**Additional file 1: Appendix**

## Data Availability

The datasets used and/or analyzed during the current study are available from the corresponding author on reasonable request.

## Abbreviations

AHA: American Heart Association
CI95: 95% Confidence Interval
FLS: Fundamentals of Laparoscopic Surgery
OPTIMAL: Optimizing Performance Through Intrinsic Motivation and Attention for Learning
