# Supplementary material for: Autonomy and focus of attention in medical motor skills learning: a randomized experiment
Source: BMC Med Educ. 2022 Jan 19;22:46. doi: 10.1186/s12909-021-03020-z (PMC8772150; doi:10.1186/s12909-021-03020-z)
Supplement: Supplementary file 1 — Additional file 1: Appendix [file 12909_2021_3020_MOESM1_ESM.zip › Instruction scripts_ESM.docx]

**PEG TRANSFER**

Your goal in this task is to move three wedges from one side of the board to the other. [Watch video] The wedges are lying on numbered pegs. Each wedge needs to be moved to the other peg with the same number [refer to the image]. After you pick up a wedge, you need to transfer the wedge to the other grasper before putting it down on the corresponding peg. Do not set down or drop the wedge until you lay it on the correct peg. If you drop a wedge, use the same grasper to pick it up and continue (for example, if you are holding the wedge in the right grasper and you drop it, you need to use the right grasper to pick it up). If the wedge falls and you cannot see it, move on to picking up the next wedge; there is no need to retrieve it.  You will be scored on both time and the number of times you drop a wedge, so try not to drop them.

Routine autonomy

**You need to move the wedges in a specific sequence. Take a look at the board; you have been assigned** [reads off assigned sequence, and places this written sequence in a location where the participant can see it]. **You've also been assigned to use the [red/green] wedges.**

Enhanced autonomy

**You need to move the wedges in a specific sequence, but you get to choose that sequence. Take a look at the board, and tell me what order you would like to move the wedges.** [supervisor gives option of 1-2-3 or 3-2-1, and records this choice, and places this written sequence in a location where the participant can see it] **You can also choose the color of wedge; do you want red or green?** [supervisor records this choice]

Internal focus of attention

[As they begin the task] **As you do the task, please focus or concentrate on your hands. Try to make small movements with your hands, and don't squeeze your hands too tightly. Open and close your hands just enough to pick up the wedge without dropping it.**

[*** Later, midway through the task, give feedback at least once] **Please remember to focus on your hands. Try to make only small movements with your hands. … Don't squeeze your hands any tighter than necessary.** [Supervisor can repeat these phrases if needed, but do NOT give any other feedback.]

External focus of attention

[As they begin the task] **As you do the task, please focus or concentrate on the graspers. Try to make small movements with the graspers, and don't squeeze the graspers too tightly. Open and close the graspers just enough to pick up the wedge without dropping it.**

[*** Later, midway through the task, give feedback at least once] **Please remember to focus on the graspers. Try to make only small movements with the graspers. … Don't squeeze the graspers any tighter than necessary.** [Supervisor can repeat these phrases if needed, but do NOT give any other feedback.]

**CHEST COMPRESSIONS**

[Participants will first watch a 2-minute video]

To emphasize what was said in the video: Really try to compress deep enough – at least 2 inches with a complete release. It’s also important to do it fast enough; 100 per minute is faster than most people think (try singing the song "Staying Alive" as you go). It helps to lock your fingers and elbows and push straight down in the middle of the chest.

Now you get to practice chest compressions on this manikin for 45 seconds. We will score you on the compression depth and frequency, so try to give your best.

Routine autonomy

**You have been assigned to do chest compressions from the [right / left] side.**

Enhanced autonomy

**You get to choose from which side to do chest compressions. Would you like to work from the right or the left side?** [supervisor records this choice]

Internal focus of attention

[As they begin the task] **Please concentrate on your hands. Try to push your hands down 2 inches, and then bring your hands all the way up before you push down again.**

[*** Later, **20 seconds** into the task, gives feedback once] **Remember to concentrate on your hands; try to push your hands down at least 2 inches**

External focus of attention

[As they begin the task] **Please concentrate on the manikin’s chest. Try to push the chest down at least 2 inches, and allow the chest to rise all the way up before you push down again.**

[*** Later, **20 seconds** into the task, gives feedback once] **Remember to concentrate on the manikin’s chest; try to push the chest down at least 2 inches.**
