# Supplementary material for: Autonomy and focus of attention in medical motor skills learning: a randomized experiment
Source: BMC Med Educ. 2022 Jan 19;22:46. doi: 10.1186/s12909-021-03020-z (PMC8772150; doi:10.1186/s12909-021-03020-z)
Supplement: Supplementary file 1 — Additional file 1: Appendix [file 12909_2021_3020_MOESM1_ESM.zip › Post-station Questionnaire_ESM.docx]

Please answer the following questions about the **CPR station** Study ID # ________

OPTION 1

| **How confident** are you that you can get the right depth (2 inches) on at least 70% of your chest compressions? | 0  Not confident at all | 1 | 2 | 3 | 4 | 5  Moder­ately confident | 6 | 7 | 8 | 9 | 10  Very confident |
| --- | --- | --- | --- | --- | --- | --- | --- | --- | --- | --- | --- |
|  |  |  |  |  |  |  |  |  |  |  |  |
| **How confident** are you that you can get the right depth (2 inches) on at least 90% of your chest compressions? | 0  Not confident at all | 1 | 2 | 3 | 4 | 5  Moder­ately confident | 6 | 7 | 8 | 9 | 10  Very confident |
|  |  |  |  |  |  |  |  |  |  |  |  |
| **How hard** did you have to work (mentally and physically) to accomplish your level of performance? | 0  Very low | 1 | 2 | 3 | 4 | 5 | 6 | 7 | 8 | 9 | 10  Very high |
|  |  |  |  |  |  |  |  |  |  |  |  |
| I feel good about the opportunity I had to make **choices** about my approach to this task. | 0  Strongly disagree | 1 | 2  Disagree | 3 | 4  Slightly disagree | 5 | 6  Slightly agree | 7 | 8  Agree | 9 | 10  Strongly agree |

Please answer the following questions about the **Peg transfer station** Study ID # ________

OPTION 1

| **How confident** are you that you can complete the wedge transfer without dropping any wedges? | 0  Not confident at all | 1 | 2 | 3 | 4 | 5  Moder­ately confident | 6 | 7 | 8 | 9 | 10  Very confident |
| --- | --- | --- | --- | --- | --- | --- | --- | --- | --- | --- | --- |
|  |  |  |  |  |  |  |  |  |  |  |  |
| **How confident** are you that you can complete the wedge transfer in less than 60 seconds without dropping any wedges? | 0  Not confident at all | 1 | 2 | 3 | 4 | 5  Moder­ately confident | 6 | 7 | 8 | 9 | 10  Very confident |
|  |  |  |  |  |  |  |  |  |  |  |  |
| **How hard** did you have to work (mentally and physically) to accomplish your level of performance? | 0  Very low | 1 | 2 | 3 | 4 | 5 | 6 | 7 | 8 | 9 | 10  Very high |
|  |  |  |  |  |  |  |  |  |  |  |  |
| I feel good about the opportunity I had to make **choices** about my approach to this task. | 0  Strongly disagree | 1 | 2  Disagree | 3 | 4  Slightly disagree | 5 | 6  Slightly agree | 7 | 8  Agree | 9 | 10  Strongly agree |
